# Patterns of Expression of Purinergic Receptor P2RY12, a Putative Marker for Non-Activated Microglia, in Aged and Alzheimer’s Disease Brains

**DOI:** 10.3390/ijms21020678

**Published:** 2020-01-20

**Authors:** Douglas G. Walker, Tiffany M. Tang, Anarmaa Mendsaikhan, Ikuo Tooyama, Geidy E. Serrano, Lucia I. Sue, Thomas G. Beach, Lih-Fen Lue

**Affiliations:** 1 Molecular Neuroscience Research Center, Shiga University of Medical Science, Seta-Tsukinowa-cho, Otsu 520-0072, Japan; anarmaa@belle.shiga-med.ac.jp (A.M.); kinchan@belle.shiga-med.ac.jp (I.T.); 2 Neurodegenerative Disease Research Center, Arizona State University, Tempe, AZ 85287, USA; ttang@pennstatehealth.psu.edu (T.M.T.); lih-fen.lue@bannerhealth.com (L.-F.L.); 3 Civin Neuropathology Laboratory, Banner Sun Health Research Institute, Sun City, AZ 85351, USA; geidy.serrano@bannerhealth.com (G.E.S.); lucia.sue@bannerhealth.com (L.I.S.); thomas.beach@bannerhealth.com (T.G.B.)

**Keywords:** activation phenotypes, microglia, neuroinflammation, immunohistochemistry, temporal cortex, Alzheimer’s disease, amyloid

## Abstract

Neuroinflammation is considered a key pathological process in neurodegenerative diseases of aging, including Alzheimer’s disease (AD). Many studies have defined phenotypes of reactive microglia, the brain-resident macrophages, with different antigenic markers to identify those potentially causing inflammatory damage. We took an alternative approach with the goal of characterizing the distribution of purinergic receptor P2RY12-positive microglia, a marker previously defined as identifying homeostatic or non-activated microglia. We examined the expression of P2RY12 by dual-color light and fluorescence immunohistochemistry using sections of middle temporal gyrus from AD, high plaque and low plaque non-demented cases in relation to amyloid beta (Aβ) plaques and phosphorylated tau, markers of pathology, and HLA-DR, IBA-1, CD68, and progranulin, microglial phenotype markers. In low plaque cases, P2RY12-positive microglia mostly had non-activated morphologies, while the morphologies of P2RY12-positive microglia in AD brains were highly variable, suggesting its expression could encompass a wider range of phenotypes than originally hypothesized. P2RY12 expression by microglia differed depending on the types of plaques or tangles they were associated with. Areas of inflammation characterized by lack of P2RY12-positive microglia around mature plaques could be observed, but many diffuse plaques showed colocalization with P2RY12-positive microglia. Based on these results, P2RY12 expression by microglia should not be considered solely a marker of resting microglia as P2RY12 immunoreactivity was identifying microglia positive for CD68, progranulin and to a limited extent HLA-DR, markers of activation.

## 1. Introduction

Alzheimer’s disease (AD) is the leading cause of dementia, currently affecting an estimated 47 million people worldwide, but this number will increase unless effective treatments are discovered [1]. Since the identification of strongly immunoreactive major histocompatibility class II HLA-DR (MHC-II)-positive microglia associated with AD pathological structures [2,3], neuroinflammation is considered a prominent feature of AD pathology [4,5]. These early studies established the hypothesis that inflammatory responses to extracellular Aβ plaques and neurofibrillary tangles might be accelerating neurodegeneration through the production of toxic inflammatory cytokines, reactive oxygen species and enzymes [6,7]. Microglia, the brain-resident macrophages, are considered the main source of these molecules. These studies suggested that anti-inflammatory agents might be effective in slowing disease progression [8], but clinical trials of anti-inflammatories have generally shown no protective effect for AD subjects [9].

There is now greater appreciation of the complexity of microglia and their many specialized functions, both pathological and reparative. Recent gene expression profiling studies of microglia isolated from human AD tissue or AD animal models have provided large amounts of data on microglial properties and identified potentially new phenotypic markers for studying microglia in disease [10,11,12,13]. These and other studies have consistently identified the purinergic adenosine diphosphate/triphosphate (ADP/ATP)) receptor P2RY12 as a significant marker for non-activated/homeostatic microglia (examples: [10,12,14,15,16]). Increased understanding of neuroinflammation will come from further classification of microglia for expression of these newly identified functional markers in human AD brain tissues. Microglial markers studied in human brains only represent a small number of potential targets. The most widely-used markers in neuropathology studies of human brains have been MHCII protein HLA-DR and ionized calcium-binding adaptor molecule (IBA-1). Increased expression of HLA-DR by microglia in AD gray matter has been consistently observed, but the specificity, significance or mechanism for this is unclear, while IBA-1 identifies all microglia and does not discriminate between phenotypes of microglia in human brains. Other microglial markers characterized in AD brains include CD68, a lysosomal-associated membrane protein associated with phagocytosis, CD32 and CD64, immunoglobulin Fc receptors, CD11b, colony stimulating factor-1 receptor (CSF-1R), Toll-like receptors (TLR)-2, 3 and 4, ferritin, CD163, Transmembrane Protein (TMEM)-119 [17,18,19,20,21,22,23] as well as Triggering receptor expressed on myeloid cells-2 (TREM-2) and CD33, microglial genes with genetic associations to AD [24,25].

P2RY12 is a member of the P2 purinergic family of receptors, a seven transmembrane-spanning G protein-coupled receptor that responds to ADP/ATP by increasing cell migration [26]. P2RY12 is mainly expressed by platelets and microglia [27]. Its function has been widely studied in relation to platelet activation and blood clotting, but its role in neuroinflammation requires further investigation. Microglia expressed significantly higher levels of P2RY12 than macrophages, in culture and in tissue, allowing discrimination between microglia and blood macrophages [11,28]. Activation of microglial P2RY12 by ADP/ATP promotes microglial chemotaxis towards sites of release [29]; these molecules are released in increased amounts by necrotic and apoptotic cells There was significantly reduced microglial chemotaxis and process formation in response to injury in P2RY12 gene-deficient mice [26]. Expression of P2RY12 by microglia (rodent and human) is downregulated after inflammatory stimulation. Injection of lipopolysaccharide (LPS) into rat brains resulted in rapid loss of P2RY12 immunoreactivity [16,22,26].

There have been limited numbers of studies of P2RY12 expression in microglia in human brains by immunohistochemistry. The most detailed previous study of P2RY12 microglia in human brains across different ages, brain regions and diseases showed expression early in brain development in all regions with limited decline with aging, while characterization of P2RY12-immunopositive microglia in 3 AD cases identified absence of P2RY12-positive microglia around Aβ plaques [30]. P2RY12-expressing microglia in astrocytomas were increased in low-grade but reduced in high-grade tumors [31]. Immunohistochemistry of human brain sections from multiple sclerosis cases confirmed loss of P2RY12 microglial immunoreactivity in areas associated with enhanced inflammation [22,30,32,33]. Another study in 2 AD cases showed that microglia positive for P2RY12 did not express TREM-2 [34].

In this report, we sought to determine if P2RY12 could be used for phenotyping the progression of human brain microglial changes in response to AD pathology by extending previous studies [30] by characterizing P2RY12 expression by microglia in detail in a staged series of AD and non-demented aged cases. The major findings showed that P2RY12 identified populations of microglia with features of resting microglia but also other populations of microglia. There was significant reduction in P2RY12 total protein levels in AD compared to ND cases, but significant amount of P2RY12 expression was present even in severe AD cases. We identified CD68 and progranulin expression in most P2RY12-positive microglia. In pathologically-involved brains, as P2RY12 expression identified microglia with many of the different morphologies associated with inflammatory activation, classifying P2RY12 expression as a marker of homeostatic (non-activated) microglia needs to be reconsidered.

## 2. Results

### 2.1. Patterns of Expression of P2RY12 by Microglia

The aim of this study was to determine if P2RY12 expression delineates populations of resting, non-activated or reparative microglia in human brains affected by different amounts of AD plaque and tangle pathology from microglia considered as pro-inflammatory and activated. If correct, areas of inflammation around pathological structures could be defined by the presence of P2RY12-positive microglia surrounding areas with P2RY12-negative (activated) microglia.

**Figure 1:**
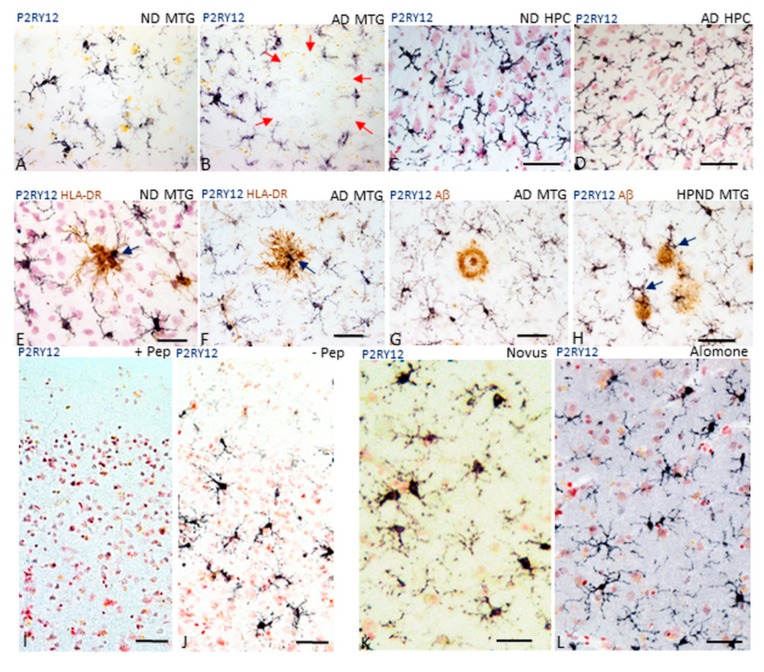
Features of P2RY12-immunoreactive microglia. (**A**,**B**). Morphology of P2RY12-immunoreactive microglia (purple) in a low plaque non-demented (LPND) case (**A**) and AD case (**B**). Sections of middle temporal gyrus (MTG) were single-stained with antibody to P2RY12. Red arrows in panel B illustrate the lack of P2RY12 immunoreactive cells in an area occupied by plaque. (**C**,**D**). P2RY12-immunoreactive microglia (purple) are a feature in hippocampus sections from non-demented (ND) case (**H**) and Alzheimer’s disease (AD) case. Section shows staining in CA2 region of hippocampus. Continued presence of P2RY12-positive microglia in AD hippocampus (**D**) was noticeable. (**E**,**F**). Double-staining of section of ND and AD case with P2RY12 (purple) and HLA-DR (brown) showed limited overlap. HLA-DR-positive microglial clusters over plaques were P2RY12-negative except for single cells observed within the cluster (arrows). (**G**,**H**). Interaction of P2RY12-immunoreactive microglia (purple) and Aβ plaques (brown). The panels show two types of interactions of P2RY12-positive microglia with plaques. Positive microglia are not present in close association with mature cored plaque (**G**), while they are present in close association with diffuse type of plaques (**H**). Specificity controls for P2RY12 staining of microglia. (**I**,**J**). Staining of representative sections with P2RY12 (Novus) antibody preabsorbed with immunizing peptide (I, +Pep) compared to staining of matched section with P2RY12 antibody non-absorbed (**J**, -Pep). (**K**,**L**). Staining of matched sections with alternative P2RY12 antibody. Same staining pattern of microglia revealed with P2RY12 (Novus) antibody (**C**) as with P2RY12 (Alomone Labs) antibody **(D**). Sections reacted with Alomone Lab P2RY12 required antigen retrieval to obtain positive staining pattern. All sections shown had been counterstained with neutral red to identify nuclei (red color). Abbreviations: ND: non-demented. AD: Alzheimer’s disease. MTG: middle temporal gyrus.—Pep: antibody without immunizing peptide. + Pep: antibody with immunizing peptide. Scale bars represent 50 μm.

Figure 1 illustrates initial observations of P2RY12-immunopositive microglia in middle temporal gyrus (MTG) and selected hippocampal sections. Figure 1A,B illustrates MTG sections stained with antibody to P2RY12 (Novus, rabbit polyclonal) from a non-demented (ND) (A) and AD (B) case. P2RY12-immunoreactive microglia with similar morphology were in sections of hippocampus from ND (Figure 1C) and AD cases (Figure 1D) (CA 2–3 region). A feature to note in Figure 1D is that although this region of hippocampus had significant neurodegenerative pathology as tangles, there was little difference in numbers and abundance of P2RY12 microglia compared to the ND case shown (Figure 1C) The distribution of P2RY12-positive microglia was noticeable with areas showing minimal staining in high pathology and AD cases (AD case shown in Figure 1B—red arrows). Areas with reduced staining were identified by accumulations of HLA-DR-positive microglia (Figure 1E,F). However, within these clusters of HLA-DR-positive microglia were isolated P2RY12-positive microglia (Figure 1E,F, blue arrows). Absence of P2RY12-positive microglia in the area around a cored Aβ immunoreactive plaque is shown (Figure 1G). However, the observation of P2RY12-expressing microglia with activated morphologies in close association with diffuse Aβ-positive plaques (high plaque non-demented (HPND) case—Figure 1H) suggested that P2RY12 expression by microglia was not restricted to non-activated microglia. These observations were the basis for further examination of P2RY12 expression by different types of microglia in this report.

**Figure 2:**
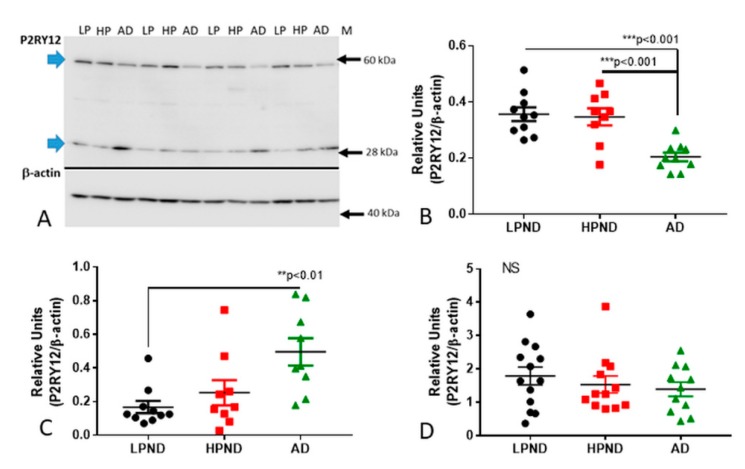
Quantitative biochemical measurements of P2RY12 protein and mRNA in human brains. (**A–C**). Western blot measurements of P2RY12 levels in MTG samples from LP, HP and AD brains. (**A**). Representative western blot image of P2RY12 polypeptide of MTG protein extracts identified with Novus antibody. Blots were normalized for levels of β actin. (**B**). Scatter plot showing individual P2RY12 expression levels. Significant decrease in protein levels of 58 kDa full-length P2RY12 band in AD (green shapes) compared to LPND (black) and HPND (red) cases. Chart indicates mean + Standard error of mean (SEM). Statistical analysis by one-way ANOVA with Tukey post-hoc test (F_2,26_ = 11.54, *p* < 0.001). (**C**). Scatter plot showing significant increase in protein levels of 30 kDa cleaved P2RY12 band in AD cases compared to LPND cases. Statistical analysis by one-way ANOVA with Tukey post-hoc test (F_2,26_ = 5.649, *p* < 0.01). Bar chart indicates mean + SEM. (**D**). Scatter plot showing expression levels of P2RY12 mRNA (normalized for β actin mRNA) in MTG samples. Lack of significant difference in expression of P2RY12 mRNA between LPND, HPND, and AD cases (MTG set 2). Samples measured by real time polymerase chain reaction. Statistical analysis by one-way ANOVA with Tukey post-hoc test (F_2,32_ = 1.031, *p* >0.05). Abbreviations: LPND: low plaque non-demented. HPND: high plaque non-demented. AD: Alzheimer’s disease. NS: non-significant. * *p* < 0.05, ** *p* < 0.01,*** *p* < 0.001.

Antibody validation was carried out to confirm that the observed immunostaining represented P2RY12 expression by microglia. Firstly, absorption of the Novus antibody with its immunizing peptide was carried out. Preincubation of diluted antibody with this 40 amino acid-recombinant peptide resulted in absence of microglial staining (Figure 1I—absorbed + PEP) compared to staining with non-absorbed antibody (Figure 1J, -PEP). The second validation step was to demonstrate the same microglial staining pattern using an independent P2RY12 antibody (Alomone Labs) produced against a different immunizing sequence. Both antibodies produced similar immunoreactivity patterns in sections from a low plaque non-demented (LPND) case (Figure 1K: Novus, Figure 1L: Alomone). To demonstrate immunoreactivity with the Alomone antibody, sections required antigen retrieval (80 °C, 30 min, 1 mM EDTA, pH 8.0), which was not required for the Novus antibody. The third stage was western blot analyses using brain protein extracts to demonstrate that the Novus antibody could detect a P2RY12 polypeptide of approximately 58 kDa, (representative western blots are shown in Figure 2A). This antibody also identified a polypeptide of approximately 30 kDa, a presumptive cleavage fragment of the full-length P2RY12 polypeptide. Although a number of different molecular weights for P2RY12 have been shown depending on cell source and antibody, a 58 kDa polypeptide is consistent with previous observations.

### 2.2. Continued Expression of P2RY12 in MTG Brain Samples with Increasing Pathology and AD: Biochemical Measurements

Increased microglial activation and pro-inflammatory cytokines have long been considered a feature of AD. If widespread throughout the AD brain, one would expect levels of P2RY12 to be very low in severe AD cases if expression was restricted to non-activated microglia. We measured the levels of P2RY12 expression in MTG sections from brains with increasing amounts of plaque pathology (Table 1, set 2). Western blot measurements of levels of P2RY12 polypeptides were made in protein samples from LPND (*n* = 10), HPND (*n* = 9) and AD cases (*n* = 9) (representative western blot–Figure 2A). Protein extracts were not available from 6 cases used in immunohistochemistry. The Novus P2RY12 antibody detected two polypeptide bands in brain samples, one of approximately 58 kDa (considered to represent full-length P2RY12) and one of approximately 30 kDa. Semi-quantitative measurements of band intensities, normalized for β-actin levels, showed significant decreased levels of 58 kDa polypeptide in AD cases (Figure 2B) but increased levels of the 30 kDa band (Figure 2C). Spearman non-parametric correlation analysis between levels of 58 kDa polypeptide and plaque and tangle scores showed significant negative correlation (P2RY12 (58 kDa)/β-actin levels compared to plaque scores; r = −0.503, *p* = 0.0039 (F_1,29_ = 7.618, *p* < 0.001): compared to tangle scores; r = −0.612, *p* = 0.0002 (F_1,29_= 24.06, *p* < 0.0001). As the Novus antibody was raised against an intracellular C-terminal peptide sequence of P2RY12, the 30 kDa bands could represent accumulations of P2RY12 after proteolytic cleavage. Its functional significance is unclear. Measurement of P2RY12 mRNA expression in cDNA derived from a similar but separate group of MTG samples (Table 1, set 3) showed non-statistically significant decrease in expression in the AD cases (Figure 2D). These results showed that significant amounts of microglial expression of P2RY12 mRNA and protein were still occurring in AD brains.

### 2.3. Patterns of Expression of P2RY12-Positive Microglia and Amyloid Beta Plaques

**Figure 3:**
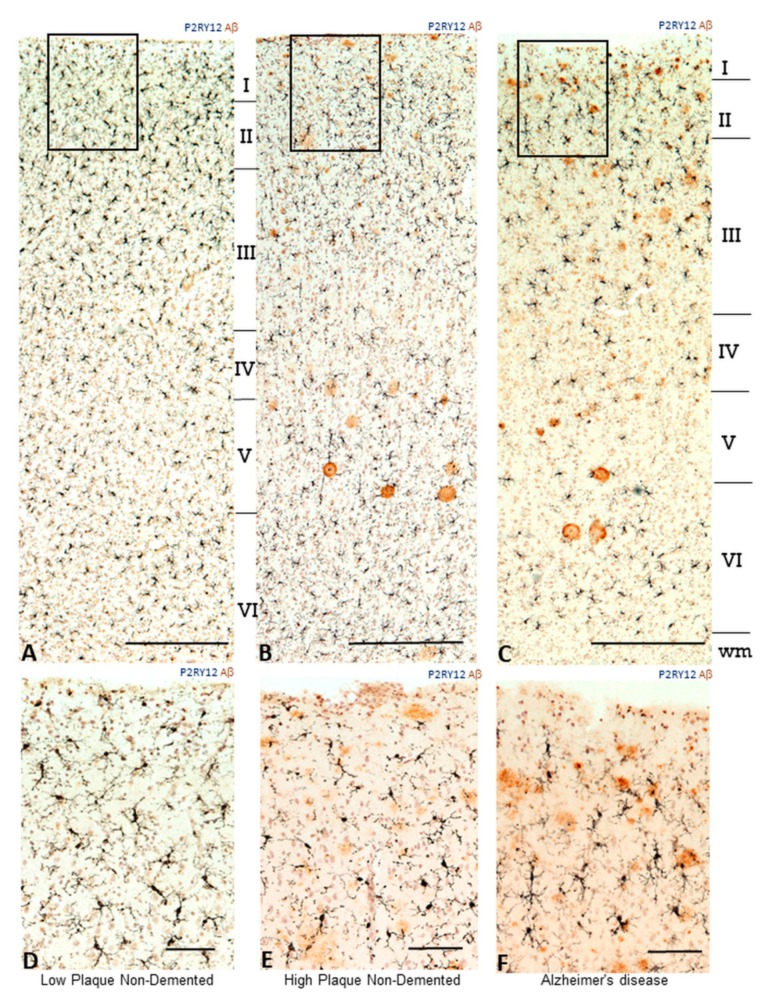
Distribution of P2RY12 microglia within cortical layers in relation to amyloid beta plaques in pathologically staged samples (**A**–**C**). Lower magnification photomicrographs showing the changes in P2RY12 microglia distribution compared to Aβ plaques within cortical layers of MTG. Sections from low plaque, high plaque and AD cases stained for P2RY12 (purple) and Aβ (brown). Sections were counterstained with neutral red to identity cellular morphology. Scale bars represent 200 μm. (**D**–**F**). Higher magnification photomicrographs of the areas in panels (**A**–**C**) indicated by frames. Scale bars represent 50 μm.

When considering features of microglial activation in AD, one feature not generally considered is the anatomical distribution of microglia within brain structure. The features of neuronal architecture throughout the cortex results in different patterns of pathological development and different phenotypes of microglia. Figure 3A–C illustrate the distributions and morphology of P2RY12-positive microglia between disease groups, with boxed areas of layer I and II for each of these sections shown at higher magnification (Figure 3D–F). These areas appeared to have high expression of P2RY12 in AD cases. Sections were double-stained for P2RY12 (purple) and Aβ brown). In the HPND case illustrated (Figure 3B,E), diffuse-type Aβ plaques are observable, particularly in layers I and II (panel E), while in the AD case, the plaques are more consolidated. The morphologies of P2RY12-positive microglia varied, particularly in HPND and AD cases, and many of the plaques had associated P2RY12-positive microglia with activated morphologies. Due to the shrinkage of cortical layers in AD, the layers in LPND and HPND sections are slightly wider; white matter regions in Figure 3A (LPND) and Figure 3B (HPND) are not visible.

### 2.4. Immunohistochemical Measurements of P2RY12 Expression

**Figure 4:**
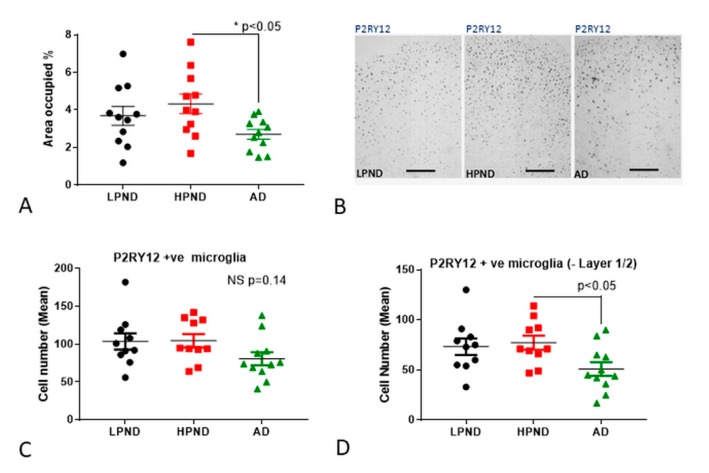
Quantitative measurements of P2RY12 immunoreactive structures in human brain tissue sections. (**A**,**B**). Measurements of area occupied of P2RY12 immunoreactivity in LPND (*n* = 11) (black), HPND (*n* = 11) (red) and AD (*n* = 12) (green) cases. (**A**). Sections were imaged at 4x magnification (three random areas/cases) and area occupied in thresholded images measured using Image J software. Results show significant decrease between HPND and AD cases. Statistical analysis by one-way ANOVA with Tukey post-hoc test (F_2,29_ = 3.903, *p* < 0.05). (**B**). Representative images of low magnification images of LPND, HPND and AD used for measurements show distribution of P2RY12 immunoreactive microglia. Scale bars represent 400 µm. (**C**). Numbers of P2RY12 immunoreactive microglia in all cortical layers. Scatter plot showing the estimated total mean number of microglia in cortical layers I–VI. Individual points represent the mean of total numbers from three separate measures for each slide. The numbers of microglia/2 mm^2^ field were counted. Results show insignificant decline in mean number of P2RY12-positive microglia in AD cases. Statistical analysis by one-way ANOVA with Tukey post-hoc test (F_2,29_ = 1.704, *p* = 0.14). (**D**). Numbers of P2RY12 immunoreactive microglia in cortical layers III-VI. Scatter plot showing the estimated total mean number of microglia in cortical layers III-VI. The numbers of microglia counted in Layers I and II were subtracted from the total. Individual points represent the mean of total numbers (except Layers I and II) from three separate measures for each slide. The numbers of microglia/2 mm^2^ fields were counted. Results show significant decline in mean number of P2RY12 positive microglia in AD cases. Statistical analysis by one-way ANOVA with Tukey post-hoc test (F_2,29_ = 3.201, *p* < 0.05). **Abbreviations**: LPND: low plaque non-demented. HP: high plaque non-demented. AD: Alzheimer’s disease. ND: non-demented. NS: non-significant.

Measurement of the occupied areas of P2RY12 immunoreactivity in a complete series of MTG sections was carried out. These sections were imaged at low magnification to include all cortical layers and analyzed using ImageJ software. There was a small but significant decrease in mean occupied area of P2RY12 immunoreactivity in AD cases (Figure 4A). Representative low-magnification images of sections used for measurements are shown (Figure 4B). Determining the numbers of P2RY12 immunoreactive microglia in stained sections in a defined area using a microscope eyepiece reticule was also carried out. These were counted in five individual fields through the cortical laminar columns (layer I and II, layer III, layer IV, layer V and layer VI), and three separate columns from each section. Mean total values for all laminar showed no significant difference between disease groups (Figure 4C), but we observed a redistribution of P2RY12-positive microglia into layers I and II in AD cases. As shown in Figure 3F, this area contained significant amount of Aβ. When cell counts for layer I and II were excluded from the analyses, the decreased number of P2YR12-positive microglia in the other layers in the AD cases was statistically significant (*p* < 0.05) (Figure 4D).

### 2.5. P2RY12 Expression and Microglial Morphology

**Figure 5:**
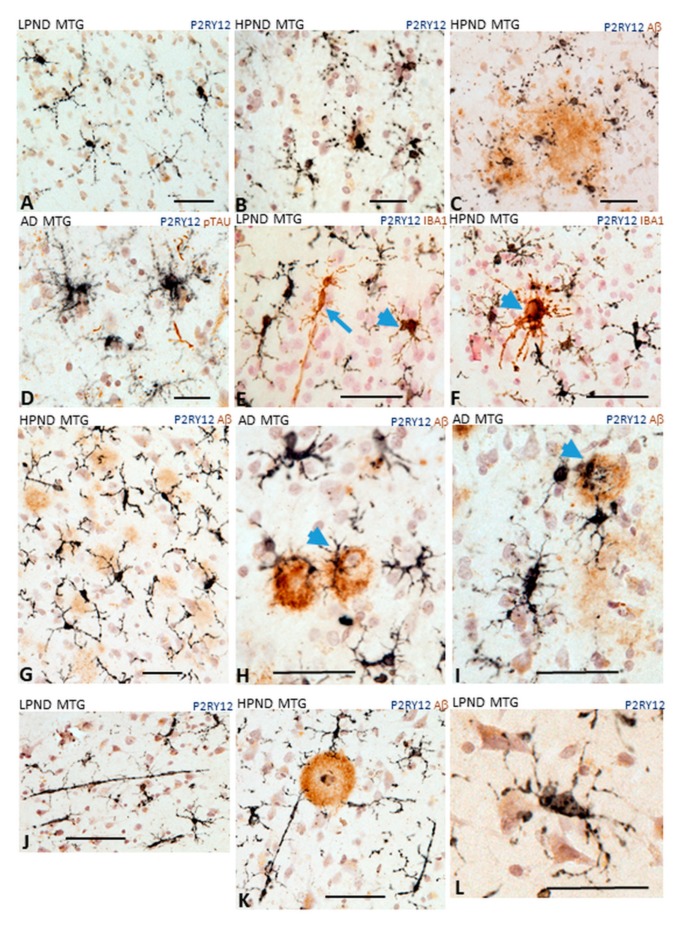
Different microglial morphologies associated with P2RY12 expression. Representative immunohistochemistry results of tissue sections stained to identify P2RY12 (purple) alone and Aβ (brown), IBA-1 or phosphorylated tau. (**A**). Ramified microglia in LPND case. (**B**,**C**). Microglia with fragmented morphology in HPND cases. (**C**). Fragmented microglia associated with diffuse Aβ plaques. (**D**). P2RY12 microglia with tufted morphology in AD case. (**E**,**F**). Colocalization of P2RY12 and IBA-1. (**E**). Rod shaped IBA-1-positive microglia (brown arrow) with minimal P2RY12 immunoreactivity. P2RY12-positive, IBA-1-positive microglia (arrowheads (**E**,**F**)). All P2RY12 immunoreactive microglia showed some IBA-1 immunoreactivity. (**F**). IBA-1 positive cluster surrounded by P2RY12 microglia. (**G**–**I**). Different morphologies of P2RY12-positive microglia interacting with Aβ plaques. (**G**). P2RY12-positive microglia with long processes interacting with diffuse plaques in HPND case. (**H**,**I**). P2RY12-positve microglia with activated morphologies (large cell bodies, short processes) interacting with dense Aβ plaques. (**J**,**K**). P2RY12-positive rod-shaped microglia in LPND (**J**) and HPND (**K**) sections. (**L**) P2RY12-positive microglial processes show interactions with neurons. Scale bars represent 50 μm.

The morphology of microglia has been considered to reflect their activation states, but these types of classifications have limitations especially in aged human brains [35,36]. The typical ramified appearance of resting microglia positive for P2RY12 in layer III of LPND case is shown (Figure 5A). Other morphologies can be observed, including dystrophic (fragmented) (Figure 5B,C) and “tufted” microglia (Figure 5D). It was observed that all P2RY12-positive microglia showed immunoreactivity for IBA-1 (purple/brown colocalization), but there were IBA-1-positive/P2RY12-negative (arrowhead) or IBA-1-positive/P2RY12-weakly positive (arrow) (Figure 5E—LPND case). Figure 5F shows the appearance of a cluster of IBA-1-positive/P2RY12-negative activated microglia (arrowhead) surrounded by strongly stained P2RY12-positive microglia. Considering the interaction of P2RY12-positive microglia with Aβ plaques, variable responses were seen. Figure 5G shows that most of the diffuse plaques (brown) in an HPND case had associated P2RY12-positive microglia (purple) with extended processes. Figure 5H,I from AD cases show microglia with more activated morphologies (shorter processes and enlarged cell bodies) interacting with more mature plaques (arrowheads). Another frequent observation was the presence of large P2RY12-positive rod-shaped microglia (Figure 5J,K), with some directly interacting with plaques (Figure 5K). One other noticeable feature seen in most cases was that many P2RY12-positive microglia, especially those with ramified processes, showed close interactions of processes with neurons (Figure 5L).

### 2.6. Confocal Microscopy Localization of P2RY12 with HLA-DR, CD68 and Progranulin in Brain Microglia

To follow up observations, further investigations of the phenotypes of P2RY12-positive microglia using antibodies to activation markers HLA-DR, CD68 and progranulin were carried out using multicolor laser confocal microscopy to demonstrate cellular colocalization.

**Figure 6:**
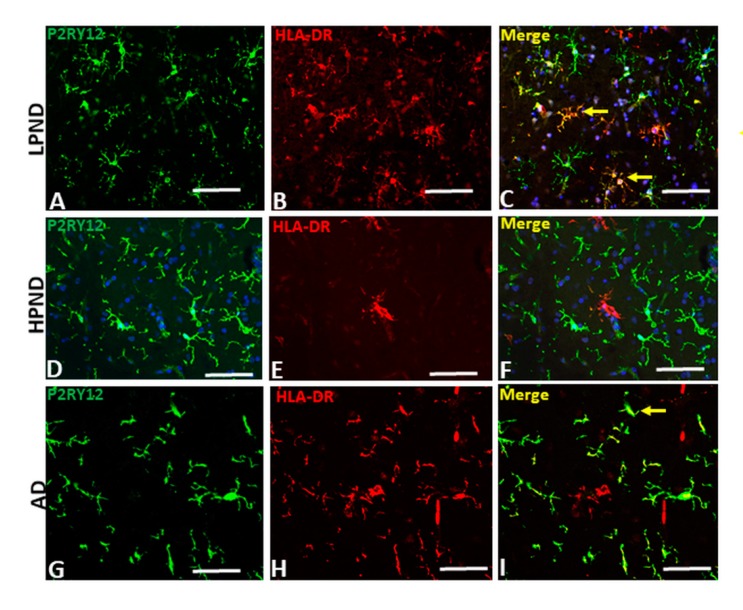
Confocal microscopy of P2RY12 and HLA-DR positive microglia in pathologically staged cases. (**A**–**I**) Images of P2RY12 (green), HLA-DR (red) and merged (yellow) with DAPI (blue) in MTG of LPND (**A**–**C**), HPND (**D**–**F**) and AD case (**G**–**I**) to show the distribution of P2RY12 and HLA-DR immunoreactivity. Examples of colocalization (yellow arrows) are shown in Merge image. Scale bar represents 50 μm.

In LPND cases with fewer HLA-DR positive microglia, those present showed colocalization with P2RY12 immunoreactivity (example: Figure 6A–C). In HPND cases, with greater numbers of HLA-DR positive microglia cases, there was less colocalization between P2RY12 (green) and HLA-DR (red) immunoreactivity (Figure 6D–F). Similarly, in AD sections (Figure 6G–I), separation of P2RY12 and HLA-DR immunoreactivity can be observed though small amounts of P2RY12 immunoreactivity with HLA-DR (Figure 6I—yellow arrow) were present in some cells.

**Figure 7:**
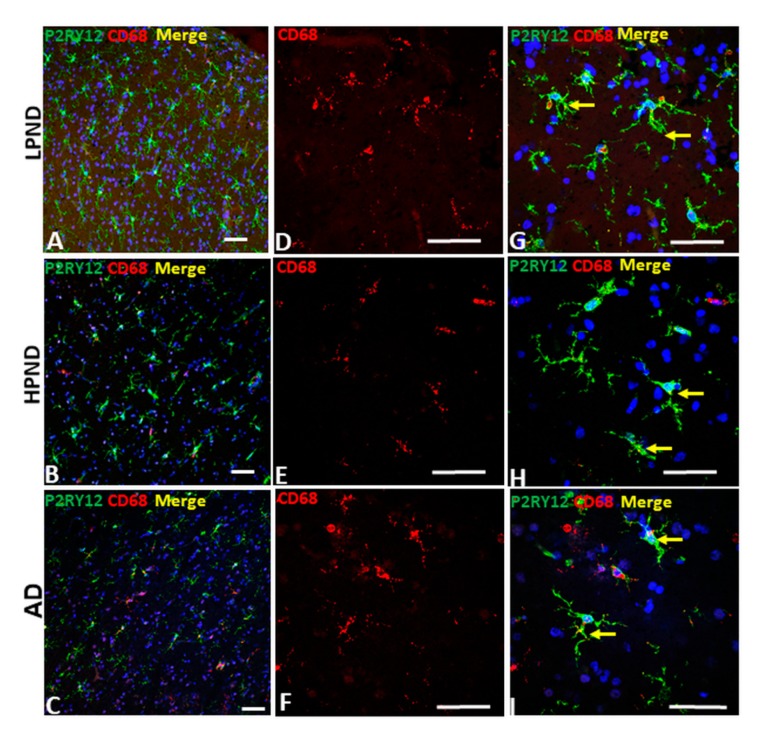
Confocal microscopy of P2RY12 and CD68-positive microglia in pathologically staged cases. **A**–**C).** Low magnification merged images of P2RY12 (green), CD68 (red) and DAPI (blue) in MTG of LPND (**A**), HPND (**B**) and AD cases (**C**) to show the distribution of P2RY12 and CD68 immunoreactivity through cortical layer. Scale bars represent 50 μm. (**D**–**F**). Higher magnification merged images of CD68 (red) in MTG of LPND (**D**), HPND (**E**) and AD cases (**F**) to show the distribution of CD68 immunoreactivity. Similar amounts of CD68 immunoreactivity was present in each disease group. Scale bars represent 50 μm. (**G**–**I**). Merged images of P2RY12 (green) with the CD68 (red) images shown in (**D**,**E**)) with DAPI (blue). Scale bars represent 50 μm.

**Figure 8:**
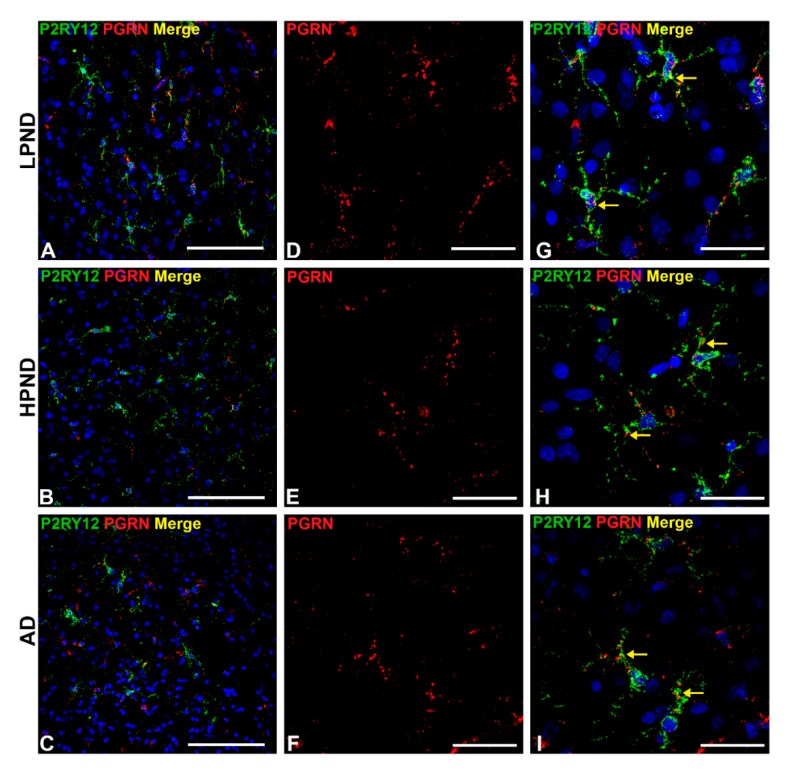
Confocal microscopy of P2RY12 and progranulin-positive microglia in pathologically staged cases. (**A**–**C**). Low magnification merged images of P2RY12 (green), progranulin (PGRN) (red) and DAPI (blue) in MTG of LPND (**A**), HPND (**B**) and AD cases (**C**) to show the distribution of P2RY12 and PGRN immunoreactivity through cortical layers. Scale bar represents 50 μm. (**D**–**F**). Higher magnification images of PGRN (red) in MTG of LPND (**D**), HPND (**E**) and AD cases (**F**) to show the distribution of immunoreactivity. Similar amounts of PGRN immunoreactivity was present in each disease group. Scale bar represents 50 μm. (**G**–**I**). Merged images of P2RY12with PGRNimages shown in (**D**–**F**) with DAPI (blue) showing expression in same cells (yellow arrows). Scale bar represents 25 μm.

Similar analyses were carried out comparing co-expression by microglia in LPND, HPND and AD MTG sections for P2YR12 and CD68, the monocyte-specific phagocytic lysosomal marker (Figure 7), and with progranulin, another lysosomal-associated marker, (Figure 8). Both proteins have been considered as markers of activated microglia. We had hypothesized that there would be a clear discrimination between P2RY12 and CD68 staining to distinguish between resting and phagocytic microglia, but this was incorrect. We observed that most P2RY12 positive microglia were also positive for CD68. The distribution of P2RY12 and CD68 in top cortical layers are shown in Figure 7A–C at low-magnification. Higher magnification images of these cases show that there was strong CD68 staining in low pathology cases (Figure 7D—red CD68 alone and Figure 7G—merged images of P2RY12 and CD68). There was no noticeable increase in CD68 intensity in HPND and AD cases (Figure 7E,F), but there were more CD68 positive cells with limited P2RY12 staining (Figure 7I) in AD cases.

Analyses of expression of progranulin in P2RY12-positive microglia in staged samples were also carried out. Progranulin is a multi-functional protein with anti-inflammatory, growth factor and lysosomal regulatory properties. Similar to CD68, microglial expression of progranulin has been associated with activated microglia [37]. The representative images shown in Figure 8 show that most P2RY12-positive microglia, whether in LPND, HPND or AD MTG sections, are also positive for progranulin. The lower magnification images (Figure 8A–C) show the distribution of P2RY12 and progranulin-positive microglia through sections of Layer II and III. All progranulin immunoreactivity was associated with cells showing different amounts of P2RY12 immunoreactivity. Although progranulin immunoreactivity can be detected in neurons with the antibody used, this was mainly detectable only in large pyramidal neurons in Layer V and not in the layers shown in this figure [38]. Higher magnifications images show progranulin immunoreactivity alone (Figure 8D–F), and merged images combined with P2RY12 show that all P2RY12 microglia appeared to be progranulin positive (Figure 8G–I).

### 2.7. Patterns of Expression of P2RY12-Microglia with Different Types of Plaques in Pathologically Staged Cases

**Figure 9:**
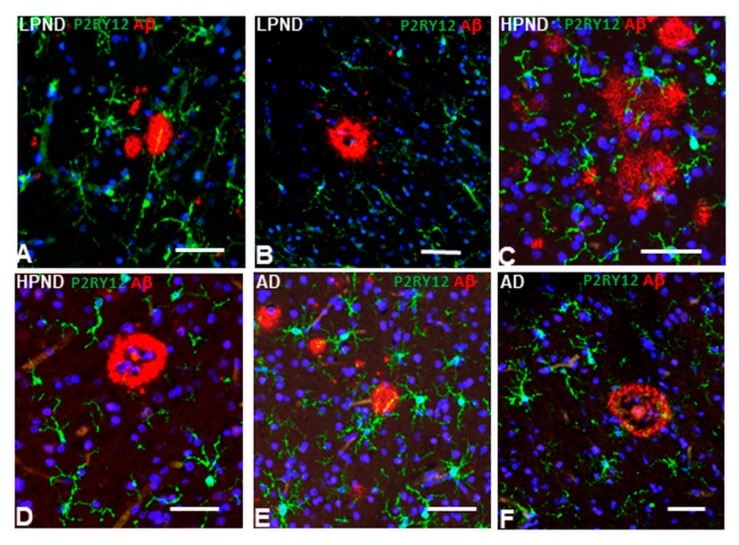
Interaction of P2RY12-positive microglia with different types of Aβ-positive plaques in pathologically-staged cases. (**A**–**F**). P2RY12-positive microglia (green) interacting with Aβ-positive diffuse-type plaques (red) (**A**,**C**,**E**), in LPND (**A**), HPND (**C**), and AD (**E**), but not with mature-type cored plaques (**B**-LPND) (**D**-HPND) (**F**-AD). Scale bars represent 50 μm.

Confocal microscopy with multi-layered images of P2RY12-immunoreactive microglia with Aβ provide additional information on their interactions. We had earlier observed that there were two types of P2RY12-positive microglia having interactions with Aβ plaques. Those surrounding plaques and those interacting with plaques. It might be assumed that all Aβ plaques would activate the microglia in a proinflammatory manner resulting in downregulation of P2RY12 expression. Figure 9 illustrates that P2RY12-positive microglia interact with diffuse-like, non-cored amyloid (earlier plaques) (Figure 9A—LPND case: Figure 9C—HPND case: Figure 9E—AD case), while the cored plaques had zones without P2RY12-expressing microglia (Figure 9B—LPND: Figure 9D—HPND: Figure 9F—AD).

### 2.8. Patterns of Expression of P2RY12-Positive Microglia and Phosphorylated Tau-Positive Tangles

**Figure 10:**
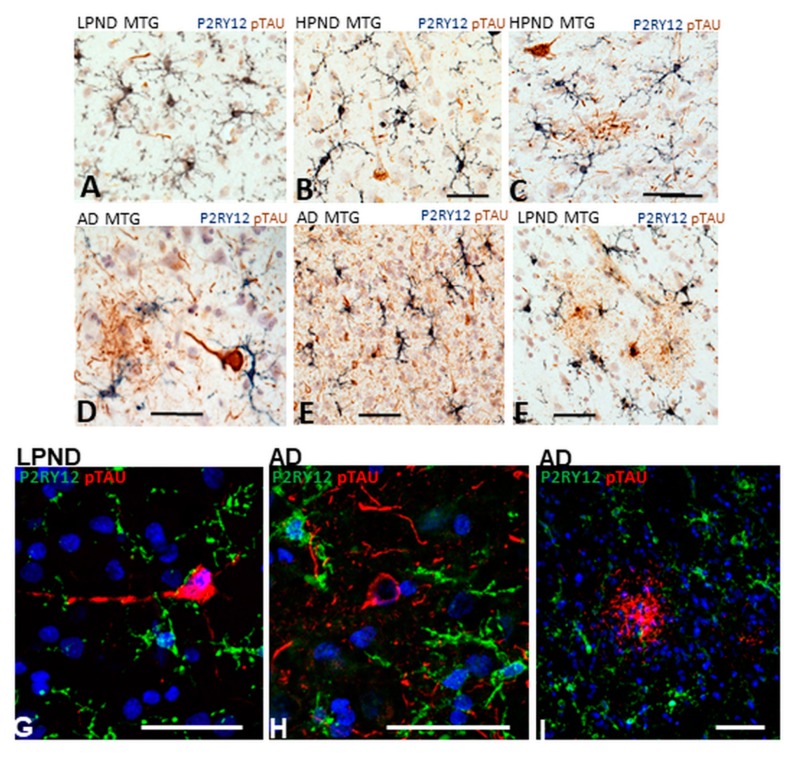
Features of P2RY12-positive microglia interacting with phosphorylated tau-containing structures. (**A–F**). Dual-color DAB enzyme histochemistry illustrating different features of P2RY12-positive microglia (purple) with phosphorylated tau-positive structures (brown). Abbreviations: LPND; low plaque non-demented. HPND; high plaque non-demented. AD; Alzheimer’s disease. MTG; middle temporal gyrus. Scale bars represent 50 μm. (**G**–**I**). Dual-color laser confocal histochemistry showing interaction of P2RY12 positive microglia with early intracellular tangle in LPND case (**G**). Features of P2RY12-positive microglia interacting with mature tangle (**H**) and neuritic plaque (**I**) in AD cases. Scale bars represent 50 μm.

Neurofibrillary tangles and dystrophic neurites are also a hallmark pathological feature of AD pathology. In this study, we identified them using antibody AT8 that recognizes phosphorylated forms of tau (serine 202/threonine 205)(pTAU) that accumulate in tangles and neurites. The cases studied had been staged based on plaque not tangle pathology. There was little difference in tangle scores between LPND and HPND cases, but a large increase in the AD cases (Table 1-set 1). AT8 staining was only prominent in the AD cases. A complete series of sections in this study were double-stained using enzyme histochemistry for P2YR12 (purple) and AT8 (brown), and a subset of these also examined by confocal microscopy. Figure 10A and 10B shows P2RY12-immunoreactive microglia interacting with sparse pTau-positive neurites (Figure 10A) and surrounding an early-stage intracellular tangle (arrow) (Figure 10B). Figure 10, panels C–E show features of P2RY12 immunoreactive microglia surrounding different AT8-immunoreactive structures. It can be seen that these microglia do not have the morphology of resting microglia. This is particular noticeable in Figure 10D with a microglia closely interacting with a tangle-containing neuron. A frequent observation in regions with heavy density of AT8 staining were the small numbers of microglia that were strongly immunoreactive for P2RY12 (Figure 10E). A rare feature observed in only one of our LPND case was the presence of AT8-positive glial cells (brown) (Figure 10F) with closely-associated P2RY12-positive microglia. Overall, the AT8-positive tangled structures did not appear to provide the inflammatory stimuli to cause downregulation of P2RY12 expression. Using confocal microscopy, Figure 10G shows an early intracellular tangle with an intact nucleus in a LPND case surrounded by P2RY12 microglia. More mature tangles and tangled neurites (Figure 10H) in AD cases did not have directly interacting P2RY12-positive microglia. Based on the characteristic morphology, the AT8-positive structure in Figure 10I is considered to represent a neuritic plaque, an accumulation of phosphorylated tau-containing neurites associated with an Aβ plaque.

### 2.9. In Vitro Analysis of P2RY12 Expression by Human Microglia

**Figure 11:**
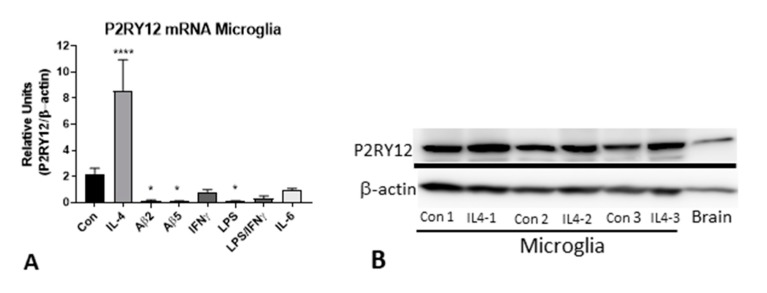
Expression of P2RY12 mRNA and protein by in vitro cultures of human microglia. (**A**). Interleukin-4 stimulates P2RY12 mRNA expression. Bar chart showing results real time PCR analysis for P2RY12 mRNA of human microglia stimulated with indicated agents. Results of analysis of single human microglia case (each in triplicate) and representative of other analyses. Abbreviations: Con, control unstimulated: IL-4, interleukin-4 (40 ng/mL): Aβ2 and Aββ5 (Aβ (1–42) 2 μM and 5 μM): IFNγ, interferon-γ (100 ng/mL): LPS, lipopolysaccharide (100 ng/mL): LPS/IFNγ (doses combined): IL-6, interleukin-6 (40 ng/mL). **** *p* < 0.0001. * *p* < 0.05. (**B**). Western blot of human microglia protein samples probed with antibody to P2RY12. Increased amounts of P2RY12 (58 kDa) in IL-4 treated samples.

Our brain tissue observations of P2RY12-expressing microglia have shown multiple features of these cells in relation to their activation states. To further investigate if increased P2RY12 expression represents a marker of alternative activation, cultured human brain-derived microglia were treated with interleukin-4 (IL-4), Aβ peptide and other proinflammatory agents to determine how activation affects P2RY12 mRNA expression. Samples analyzed by qPCR for P2RY12 mRNA expression showed strong induction of expression by IL-4 treatment, and downregulation to different extents with Aβ and other proinflammatory agents (Figure 11A). Induction of P2RY12 protein was also observed by western blotting in IL-4-treated microglia (Figure 11B).

## 3. Discussion

The aim of this work was to examine phenotypes of P2RY12-immunopositive microglia in aging and AD brains in relation with AD-associated pathological structures. P2RY12 has been defined as a specific marker to discriminate between microglia, with high levels of expression, and macrophages, with low levels of expression [16,30]. In addition, based on experimental findings, continued expression by microglia of P2RY12 in brain should define them as non-activated, namely those not producing proinflammatory cytokines associated with enhanced inflammation. As experimental studies have shown that proinflammatory activation of microglia resulted in significant reduction in P2RY12 expression [16,26], it was hypothesized that identifying microglia with high expression of P2RY12 compared to microglia positive for classical activation markers but with low to negative expression of P2RY12 would provide a means of identifying areas of active inflammation in brain tissue [30]. The findings of this work showed that microglial expression of P2RY12 was downregulated in AD tissue samples, but immunohistochemistry identified more complex patterns of increased P2RY12 expression associated with pathological structures than previously identified [30].

Recent gene expression profiling of single-cell microglia from rodent and human sources had confirmed that P2RY12 mRNA expression was associated with a non-activated phenotype and expression was downregulated with progression of disease [10,14,15]. However, our initial observations in human brains suggested that this might not cover all features of P2RY12 expression by microglia; for this reason, we sought to provide detailed characterization of P2RY12 microglia in human aged and AD brains. The importance of microglial phenotyping to identifying functional markers is now appreciated along with the need for greater numbers of markers [39]. For a number of years, the classification of microglia (and macrophages) into functional M1 and M2 groups was applied but it is now appreciated that this system does not account for the complexities of microglial phenotypes in diseased brains [40]. Recent findings have defined a phenotype of microglia designated “disease-associated microglia” (DAM), which describes a transcriptional signature first associated with response to neurodegeneration-associated molecular patterns (DAM stage 1) that progresses to a signature associated with a protective role to limit inflammation (DAM stage 2) that is coordinated by TREM-2 signaling. Downregulation of P2RY12 from homeostatic to stage 1 DAM confirms earlier findings but our cellular localization findings suggest that upregulation of P2RY12 may also be a feature associated with later stages of AD.

We have made some new findings on P2RY12 expression by microglia in human brains as part of this study that extend previous findings [30]. There were decreased levels of total P2RY12 protein of 58 kDa in brain extracts from AD cases compared to non-demented low and high pathology cases, as could be expected, but we also identified increased levels of 30 kDa P2RY12 polypeptide in AD cases. This indicates that downregulation of P2RY12 protein levels might be due to enhanced cleavage of this plasma membrane protein. The antibody we used for our study was prepared against a 40-amino acid recombinant peptide corresponding to the C-terminal cytoplasmic domain of P2RY12 (amino acids 303–342). P2RY12 is a G-protein-coupled receptor for ADP containing 7-transmembrane domains. Based on this structure, the 30 kDa polypeptide would represent a remaining cell-associated peptide that does not contain the N-terminal sequences. As P2RY12 has been reported to have multiple ADP-binding domains, it is unclear if this fragment will be biologically active for ADP binding and signal transduction. Downregulation of P2RY12 expression as a result of proinflammatory activation and upregulation as a response to IL-4 were regarded as features of alternatively activated microglia though the mechanisms for this to occur in brains is unclear as IL-4 expression has not been consistently detected in brain tissue [41]. We showed that downregulation of P2RY12 expression following inflammatory stimulation also occurred in cultured human microglia as did others [42]. The involvement of progranulin, a neuroprotective and anti-inflammatory molecule, in microglial function is still unclear. Progranulin positive microglia are found throughout brain but our findings showed P2RY12-positive microglia in all disease groups were positive for intracellular progranulin. It has been shown that IL-4 upregulates progranulin expression by cultured human microglia [43]. Based on current findings of gene regulation, one can speculate that P2RY12/progranulin positive microglia are protective rather than reactive, but further studies are required.

A previous study of P2RY12 microglia across different human brain regions and ages made similar observations as this study that most P2RY12-positive microglia were also CD68 positive [30]. CD68, a myeloid specific lysosomal-associated membrane protein associated with phagocytosis, has been considered as a microglial activation marker in a number of studies [23,44], but colocalization of CD68 and P2RY12 would suggest its involvement in normal microglial function. Two earlier studies have observed that P2RY12 was not expressed by microglia accumulating around plaques in AD brains. We also observed this for many plaques, but there were noticeable exceptions as many diffuse-like Aβ(6E10-immunoreactive) plaques had P2RY12-positive microglia interacting with them. We also observed varied morphologies for P2RY12-microglia interacting with plaques, including fragmented, tufted and rod-shaped. The study of Mildner et al. ([30]) employed a different antibody (Sigma-Aldrich HPA014518) than we used (Novus NBP2-33870), however both antibodies were produced against the same C-terminal 40-amino acid peptide sequence so should have the same properties. Detection differences in these studies could be due to tissue fixation conditions. Our study employed lightly-fixed free-floating brain sections for immunohistochemistry, while Mildner et al. employed harder-fixed paraffin-embedded sections [30]. For their study, antigen retrieval was required for all antibodies, while we found this not necessary for free-floating sections when using the Novus antibody. However, sensitivity of P2RY12 detection to fixation was observed as the alternate antibody (Alomone-APR-012) we used only worked when free-floating sections underwent antigen retrieval processing.

Although P2RY12 appears to be an excellent marker for microglia in brain, it is unclear whether expressing microglia can be classified as protective or proinflammatory. How P2RY12-mediated responses by microglia are involved in AD pathogenesis is unresolved. P2RY12-mediated chemotactic responses to ATP and ADP, which are released by damaged or dying cells, appears to be an early inflammatory response. The rapid downregulation of P2RY12 expression with proinflammatory activation would appear to function to anchor microglia at sites of inflammation. It has been proposed that downregulation of P2RY12 is accompanied by increased expression of adenosine A2 receptor, the breakdown products of P2RY12 ligands ADP/ATP [45]. A recent in vitro study of microglia demonstrated the proinflammatory consequences of inflammasome and NFκB activation by extracellular ADP activation of P2RY12 [46]. In a rodent ischemia model, blockade of microglial P2RY12 with ticagrelor, an antagonist, reduced ischemic damage by microglia by reducing their migration to sites of injury [47]. In another ischemia animal model, and with an in vitro model of oxygen–glucose deprivation (OGD), inhibition of microglia P2YR12 with clopidogrel, another antagonist, or by P2RY12 gene expression knock-down significantly reduced microglial migration and neurotoxicity [48].

**Figure 12:**
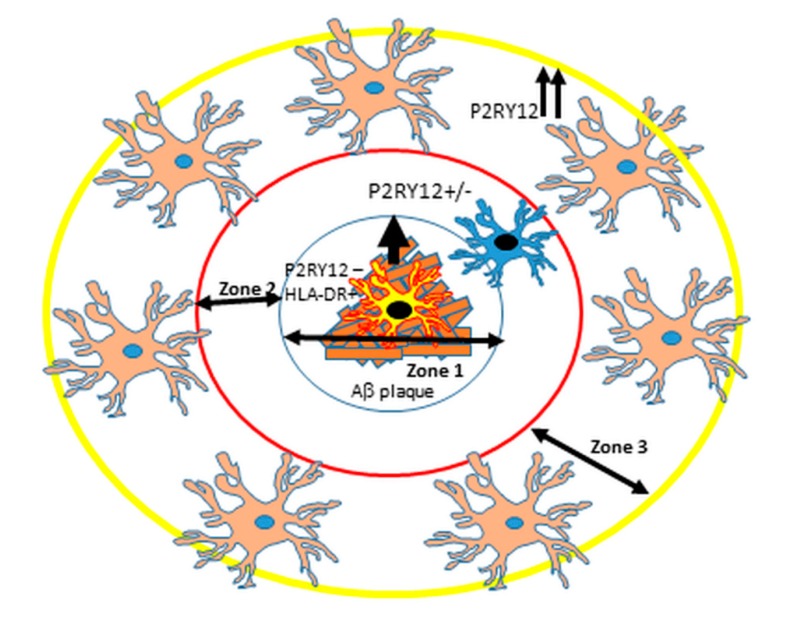
Proposed scheme of arrangement of different P2RY12-expressing microglia around Aβ plaques. Suggested scheme to describe localized areas of microglial inflammation around plaques. Zone 1: microglia interacting with mature plaques (HLA-DR high, P2RY12 negative) producing proinflammatory cytokines. Zone 2: Area adjacent to plaque with low or negative P2RY12 positive microglia. Zone 3. P2RY12 high expression in surrounding area defining the boundary between proinflammatory area (Zone 1 and 2) and non-affected area (Zone 3 and beyond). As described in this report, exceptions to this scheme were observed.

The potential for using P2RY12-positive microglia distribution to define distribution of neuroinflammation in AD or other neurodegenerative diseases is still valid, though not as clear as originally hypothesized. There has been a widely-held concept that AD neuroinflammation is widespread through affected areas, but these findings suggest it is highly localized. If one considers the scheme outlined in Figure 12, which is a representation of Figure 1E,F,G and Figure 9D,F, that activated microglia (low-negative P2RY12 microglia) associated with mature amyloid plaques are secreting cytokines and other molecules that downregulate P2RY12 expression by microglia, these will be present in the zone around the plaques (Zones 1 and 2), but not in Zone 3 where the P2RY12-positive microglia are located. With the use of laser capture microscopy and proteomics techniques, analyses of micro-dissected regions are feasible and could identify key neuroinflammatory factors associated with AD that are not represented when larger dissected pieces of brain tissue are analyzed. The feasibility of this approach has been demonstrated in proteomic analyses of micro-dissected plaques and tangles from AD brains [49,50].

## 4. Materials and Methods

### 4.1. Human Brain Tissue Samples

Human brain tissue samples used in this study were obtained from the Banner Sun Health Research Institute Brain and Body Donation Program, Sun City, Arizona, U.S.A. [51,52]. The operations of the Brain and Body Donation Program (BBDP) have received continuous approval of Institutional Review Boards (IRB). Current operations have been reviewed by Western IRB (Puyallup, WA, USA). Written informed consent for collection and use of brain and other tissues for research purposes were obtained from donors or next-of-kin. Tissue studies carried out in the U.S.A. were considered non-human subject research under federal regulations. Tissue studies carried out in Japan were approved by Shiga University of Medical Science Ethical Committee (Project Certificate no. 29-114). Demographic details of cases used in this study are summarized in Table 1.

### 4.2. Brain Tissue Preservation and Fixation

All brains were processed at autopsy in a standardized manner [52]. The median postmortem interval for autopsies in the BBDP was 3.8 h. After brain removal, the cerebellum and brain stem are separated from the hemispheres, then each brain is sectioned in a frame into 1cm thick coronal slabs. The hemispheres are divided, with the left hemisphere being frozen on dry ice for storage at −80 °C, and the right hemisphere being fixed for 48 h in buffered formalin solution. After fixation, the coronal pieces are rinsed and transferred to a phosphate-buffered solution of 15% glycerol/15% ethylene glycol as cryoprotectant. Brain regions used for subsequent studies are dissected from frozen or fixed coronal slices by experienced neuroanatomists.

### 4.3. Neuropathological Diagnosis Criteria

All donated brains received full neuropathological diagnosis including reference to pre-mortem clinical history of each case. Consensus clinical and neuropathological criteria were used to diagnose AD, Dementia with Lewy bodies (DLB) and PD in these cases [53,54]. To assess severity of AD pathology in each case, tissue sections from 5 brain regions (entorhinal cortex, hippocampus, frontal cortex, temporal cortex and parietal cortex) were stained with Thioflavin-S, Gallyas or Campbell–Switzer histological stains and assessed semi-quantitatively for the density of neurofibrillary tangles and amyloid plaques with each brain region being ranked on a scale of 0–3. By combining the measures across these 5 brain regions, assessment of AD pathology was ranked on an ordinal scale of 0–15 for plaques and tangles [55]. The two sets of cases used in this study were subdivided into non-demented low plaque (LPND) (plaque score < 6), non-demented high plaque (HPND)(plaque score 6–14) and AD with dementia (plaque score > 12).

### 4.4. Peroxidase/Diaminobenzidine Immunohistochemistry

Formaldehyde-fixed tissue sections (25 μm) from middle temporal gyrus (MTG) from 36 cases cut on a sliding microtome were used for cellular localization of purinergic receptor P2RY12 in relation with AD pathological markers, amyloid-beta (Aβ and phosphorylated tau, or markers of microglia activation (HLA-DR, and IBA-1). Sections of hippocampus from 4 cases (2 LP, 2 AD) were also used for comparison. A free-floating immunohistochemistry method was used. Tissue sections from a series of cases were rinsed three times with phosphate-buffered saline-Triton X100 (PBSTx) (0.1 M phosphate Buffer, pH 7.6, 0.137 M NaCl, 0.3% Triton X100) and pretreated with 1% hydrogen peroxide in PBSTx for 30 min to quench endogenous tissue peroxidases. For certain antibodies, antigen retrieval was carried out by heating sections in 10 mM EDTA (pH 8.0) at 80 °C for 30 min and then cooling to room temperature for 30 min. Sections were incubated free-floating at room temperature for 18 h with shaking in PBSTx with optimal dilution of antibodies. To identify localized antibodies, sections were sequentially washed three times in PBSTx for 10 min, incubated in biotinylated secondary antibody (1:1000), sequentially washed again three times in PBSTx and then incubated in preformed avidin-biotin horseradish peroxidase enzyme complex (ABC-Vector Laboratories, Burlingame, CA USA) solution (1:1000) for 1 h. Sections were then washed three times in PBSTx and two times in 50 mM Tris-HCl (pH 7.6) before incubation in peroxidase substrate. Most frequently used was nickel ammonium sulfate-enhanced diaminobenzidine as substrate to produce a purple reaction product (50 mM Tris-HCl, pH 7.6, 1% saturated nickel ammonium sulfate, 40 mM imidazole, 100 µg/mL diaminobenzidine-HCl (Dojindo, Kumamoto, Japan) and 0.0003% hydrogen peroxide). For two-color immunohistochemistry, reacted sections were rinses in PBSTx, treated with 1% hydrogen peroxide to remove residual peroxidase activity and then incubated for a second time in primary antibody overnight at room temperature. The detection procedure followed the above described protocol except the substrate used was diaminobenzidine without nickel ammonium sulfate as substrate to produce a brown reaction product (50 mM Tris-HCl, pH 7.6, 20 mM imidazole, 200 µg/mL diaminobenzidine-HCl and 0.0006% hydrogen peroxide). Reacted sections were mounted on slides, counterstained in most cases with 0.5% neutral red, dehydrated, cleared and coverslipped using Permount mounting media (ThermoFisher, Waltham, MA, USA Sections used for quantitative measurements were not counterstained.

### 4.5. Fluorescent Confocal Immunohistochemistry

Multiple-color fluorescent confocal immunohistochemistry was carried out for antibody pairs to verify cellular co-localization of antigens with P2RY12-expressing cells [19]. Tissue sections were incubated with optimal dilutions of antibodies at room temperature overnight with shaking. After three washes (10 min each) in PBSTx, sections were incubated in the dark with optimal concentrations of fluorescent-labeled secondary antibodies. Bound primary antibodies were detected with Alexa Fluor 488 (donkey anti-goat IgG), Alexa Fluor 568 (donkey anti-rabbit or anti-mouse IgG) or Alexa Fluor 647 (donkey anti-mouse IgG) (ThermoFisher, San Jose, CA, USA). After washing and mounting, sections were counterstained with Sudan Black (1% solution in 70% ethanol for 10 min) to quench tissue autofluorescence, destained with 70% ethanol, and stained with DAPI to reveal nuclei. Sections were coverslipped using antifading-hardening-fluorescent mounting media (Vector Labs, Burlingame, CA, USA). Sections were imaged using an Olympus FV1000 confocal microscope, and compiled Z-scans obtained and processed using Olympus microscope system software (Olympus Corporation, Tokyo, Japan). Some sections were imaged using a Leica SP8 confocal microscope system (Leica-Microsystems, Wetzlar, Germany).

### 4.6. Antibodies

The following primary antibodies were used in this study: P2RY12 (Novus Biologicals, Centennial, CO, USA) catalog no. NBP2-33870; rabbit, 1:1000–1:2000 used for immunohistochemistry (IHC) and western blot (WB). P2RY12 (Alomone Labs, Tel Aviv, Israel); catalog no. APR-012; rabbit, 1:200 used for IHC. P2RY12 (Abcam, Cambridge, UK); catalog no. AB83066; rabbit, 1:2000 used for WB. HLA-DR clone LN3 (Abcam); catalog no. AB80658; mouse, 1:750 used for IHC. CD68 (Biolegend, San Diego, CA, USA); catalog no 916104; mouse, used at 1:250 for IHC. Progranulin (R&D Systems, Minneapolis, MN, USA; catalog no. AF2420, goat used at 1:100 for IHC. IBA-1 (Wako, Richmond, VA, USA); catalog no. 019-19741; rabbit, 1:1000 used for IHC. Aβ clone 6E10 (Biolegend); catalog no. 803001; mouse, 1:2000 used for IHC. pTau clone AT8 (ThermoFisher, Waltham, MA, USA); catalog no. MN1020: mouse, 1:3000 used for IHC.

Secondary biotinylated-antibodies used for enzyme histochemistry and Avidin-Biotin-Complex (ABC) peroxidase were obtained from Vector Labs (Burlingame, CA, USA). Fluorescent-labeled secondary antibodies used for confocal microscopy, and horseradish peroxidase (HRP)-conjugated secondary antibodies used for western blots were obtained from ThermoFisher (Waltham, MA, USA).

### 4.7. Verification of Antibody Specificity

The majority of the immunohistochemistry studies reported in this communication were carried out with the Novus P2RY12 antibody. Peptide absorption studies were carried out using a recombinant 40-amino acid protein (NBP2-33870PEP, Novus), the immunizing peptide for the Novus antibody (NBP2-33870). P2YR12 antibody (1:1000) was mixed with 20-fold molar excess of protein for 18 h, and these materials were used to stain sections from three separate cases in parallel with non-absorbed antibody. In addition, comparisons of immunostaining patterns were carried using an independent antibody to P2RY12 (1:250, Alomone Labs), prepared against an 18-amino acid peptide sequence that did not overlap with the immunizing sequence of the Novus antibody.

### 4.8. Brain Sample Extraction and Western Blot

Brain tissues samples (middle temporal gyrus – MTG) were dissected frozen and then further trimmed so samples being analyzed primarily contained gray matter. Detergent-soluble extracts were prepared by gently sonicating each tissue sample in 5 volumes (weight to volume) of RIPA buffer (20 mM Tris-HCl, pH 7.5. 150 mM NaCl, 1% Triton X100, 1% sodium deoxycholate, 0.1% sodium dodecyl sulfate) supplemented with protease and phosphatase inhibitors (Nacalai-Tesque, Kyoto, Japan). After 30 min incubation on ice, samples were centrifuged at 15,000×*g* for 30 min. The supernatants were transferred to new tubes and total protein concentration of each extracted sample was determined using a Micro BCA assay kit (ThermoFisher, Rockford, IL, USA) with bovine serum albumin as standard. P2RY12 polypeptides were detected in MTG samples by western blots using the Novus P2RY12 antibody (1:1000). Protein samples were dissolved in SDS-sample buffer, adjusted to contain 1 μg/μL protein, denatured by heating to 90 °C for 10 min, centrifuged at 15,000×*g* for 10 min to remove insoluble material and then separated on 4–20% Tris-glycine precast gradient polyacrylamide gels (Nacalai-Tesque, Kyoto, Japan). Separated polypeptides were transferred to PVDF membranes (Millipore-EMD) using a semi-dry transfer apparatus. Membranes were blocked in 5% skimmed milk solution diluted in Tris-buffered saline with 0.1% Tween 20 (TBST), and incubated for 18 h in optimal dilutions of antibody solution in 2% milk in TBST. Membranes were washed in TBST and then reacted with HRP-labeled secondary antibody for 2 h. Membranes were reacted with Chemi-Lumi-One Super chemiluminescent substrate (Nacalai-Tesque, Kyoto, Japan) and sequential images captured using an ImageQuant LAS 4000 system (GE LifeSciences, Pittsburgh, PA, USA). Band intensities were measured using Image Studio Lite (LI-COR, Lincoln, NE, USA). All membranes were subsequently reacted with HRP-labeled antibody to β-actin (FujiFilm Wako Pure Chemicals, Osaka, Japan) at 1:15,000 for 1 h and imaged in the above-described manner. Band intensities were normalized for levels of β-actin in samples.

### 4.9. Area of P2RY12 Immunoreactivity

To measure area of P2RY12 immunoreactivity, a complete series of cases (see Table 1) were single-stained with P2RY12 antibody (Novus) using nickel-enhanced DAB to reveal dark purple reaction product. After mounting and coverslipping of sections, images at 4x magnification were captured from each section, taking four separate, random fields of gray matter for each section. Captured images were analyzed using ImageJ analysis software (NIH, Bethesda, MD, USA) (https://imageJ.net/ImageJ, version 1.52a, accessed on 5 January, 2018) to measure area of immunoreactivity occupied by reaction product on each slide. Images were converted to gray scale and thresholds adjusted to identify positively stained microglia. Mean values of area occupied between the four separate fields measured were calculated for each case, and then mean data were compiled into respective disease groups for further statistical analysis.

### 4.10. P2RY12 Immunoreactive Cell Counts

Further analyses were carried out to estimate numbers of P2RY12 immunoreactive microglia in each section. Using a 25-grid-square microscope eyepiece reticule (Nikon) and 20× objective, the numbers of microglia were counted in five consecutive areas through the cortex. Each field counted corresponded to 2 mm^2^. These areas corresponded approximately to Layer I and II, Layer III, Layer IV, Layer V, and Layer VI. For each section, three separate areas were counted where distinct cortical layers could be detected. The patterns of cortical layers was confirmed by reference to standard text. These measures allowed sampling through all cortical layers though the AD cases had thinner cortical layers. Counting criteria required the presence of a microglial cell body to be present within the area of the reticule grid. For each case, the mean total numbers of cells were calculated, and then mean and standard error of mean for all samples from each disease group.

### 4.11. Quantitative Reverse Transcription Polymerase Chain Reaction (qPCR) Analysis of mRNA Expression.

RNA was prepared from human brain tissue samples and human microglia using RNAeasy Plus-Mini kits (Qiagen, Valencia, CA, USA) according to the manufacturer’s protocol. RNA concentrations and purities were measured using a Nanodrop 1000 spectrophotometer and RNA integrity with an Agilent Bioanalyzer and RNA 6000 Nano kits (Agilent, Santa Clara, CA, USA). Samples used for qPCR had RIN values greater than 7.0. RNA from brain samples (0.5 μg) and cultured cell samples (0.2 μg) were reverse transcribed using the Quantitect reverse transcription kit (Qiagen) with genomic DNA elimination reagent according to manufacturer’s protocol. Appropriate numbers of no reverse transcriptase controls were prepared in parallel for each batch of samples. For qPCR, cDNA samples were amplified using Perfecta Fast Mix 2x reaction mixture (Quanta Biosciences, Gaithersburg, MD, USA) supplemented with 1.25 μM of Eva Green. The primers used were as follows: P2RY12 sense: AGTCCCCAGGAAAAAGGTG; P2RY12 antisense: GTTTGGCTCAGGGTGTAAGG (reference sequence NM_022788.40). Expression results were normalized with relative levels of β-actin mRNA using primer sequences: β-actin sense: TCCTATGTGGGCGACGAG. β-actin antisense: ATGGCTGGGGTGTTGAAG. QPCR was carried out using a Stratagene Mx3000p machine and abundance of gene expression quantified relative to a standard curve of pooled samples. QPCR analyses followed recommended criteria for minimum information for publication of quantitative real-time PCR experiment (MIQE) [56].

### 4.12. Human Brain-Derived Microglia Isolation and Stimulation

Human brain microglia were prepared from frontal cortex from three different donor cases for this study following our published procedures [9,13]. After 10–14 days in culture, microglia were replated into wells at 10^5^ cells/well in 12-well plates prior to stimulation. For these experiments, microglia were unstimulated or treated with interleukin (IL)-4 (20 ng/mL), aggregated Aβ42 (2 μM and 5 μM), interferon-γ (IFNγ) (20 ng/mL), lipopolysaccharide (LPS) (100 ng/mL), LPS and IFNγ combined, and IL-6 (20 ng/mL) [9]. After 24 h treatment, RNA was isolated from microglia using the above described methodology. Expression of P2RY12 mRNA in treated and untreated cells were carried out as described above for brain samples. Western blot analysis for P2RY12 protein expression by IL-4 stimulated cells was carried out with microglia from a single additional case.

### 4.13. Data Analysis

Data for relative changes in relation to disease classification were analyzed by one-way analysis of variance (ANOVA) with Newman–Keuls post-hoc test for significance between paired groups. Significant differences were assumed if *p* values of less than 0.05 were obtained. Statistical analyses were carried out using Graphpad Prism Version 7 software (Graphpad Software, La Jolla, CA, USA).

## Figures and Tables

**Table 1:** Demographic details of human brain cases used.

Set 1: Middle temporal gyrus (Immunohistochemistry)						
***Disease State (n)***	***Age***	***Sex***	***ApoE4***	***Plaques***	***Tangles***	***Braak***
LPND (*n* = 12)	85.9 ± 8.9	6M/6F	4.5%	1.3 ± 1.9	4.8 ± 2.8	I-IV
HPND (*n* = 12)	88 ± 8	4M/8F	13.6%	12.2 ± 1.6	5.4 ± 2.3	II-IV
AD (*n* = 12)	79.2 ± 5.1	7M/5F	33.3%	14.2 ± 0.8	13.8 ± 1.9	V-VI
Set 2: Middle temporal gyrus (Western blot)						
***Disease State (n)***	***Age***	***Sex***	***ApoE4***	***Plaques***	***Tangles***	***Braak***
LPND (*n* = 10)	86.3 ± 8.9	6M/4F	4.5%	1.7 ± 2.1	5.5 ± 2.3	I-IV
HPND (*n* = 9)	86.9 ±8.6	3M/6F	13.6%	12.2 ± 1.6	5.4 ± 1.9	II-IV
AD (*n* = 9)	78.2 ± 3.9	5M/4F	33.3%	14.4 ± 0.6	13.8 ± 1.9	V-VI
Set 3: Middle temporal gyrus (RNA expression)						
***Disease State (n)***	***Age***	***Sex***	***ApoE4***	***Plaques***	***Tangles***	***Braak***
LPND (*n* = 13)	85.7 ± 9.3	7M/6F	0.0%	1.2 ± 1.9	4.9 ± 2.8	I-IV
HPND (*n* = 12)	86.1 ± 6	6M/6F	12.5%	11.4 ± 2	4.7 ± 2.3	II-IV
AD (*n* = 11)	81.2 ± 3.4	8M/3F	31.8%	14.4 ± 0.7	13.0 ± 2.7	V-VI

**Abbreviations**: ApoE4: % ApoE4 alleles; Plaques: mean plaque score + SEM (scale 0–15); tangles: mean tangle score + SEM (scale demented. AD: Alzheimer’s disease 0–15); LPND: low plaque non-demented; HPND: high plaque non- demented.

## Abbreviations

P2RY12	Purinergic receptor P2Y12
LPND	Low plaque non-demented
HPND	High plaque non-demented
AD	Alzheimer’s disease
Aβ	Amyloid beta
IL	Interleukin
LPS	Lipopolysaccharide
